# *Bos taurus* and *Bison bison* conservative retrotransposon recombination products

**DOI:** 10.3389/fvets.2025.1516731

**Published:** 2025-04-30

**Authors:** Gleb Yu. Kosovsky, Galina V. Glazko, Olga I. Skobel

**Affiliations:** ^1^Department of Biotechnology, Afanas‘ev Research Institute of Fur-Bearing Animal Breeding and Rabbit Breeding, Moscow, Russia; ^2^Department of Biomedical Informatics, University of Arkansas for Medical Sciences, Little Rock, AR, United States

**Keywords:** cattle, bison, retrotransposons, microRNA, miR-30, artificial selection, domestication, RTE-BovB

## Abstract

**Background:**

Without identifying and studying the genomic characteristics associated with domestication, managing farm animal genetic resources becomes overwhelmingly difficult. Accumulated data confirm that mobile genetic elements participate in the domestication process and, in particular, generate widely abundant microRNAs.

**Methods:**

The recombination products were compared *in silico* between the long interspersed nuclear element (LINE) and the endogenous retrovirus (ERV), forming the LINE/ERV/LINE sequence, located in a closely linked conserved block of 12 genes, as well as the microRNAs formed by these recombination products in domesticated-wild pairs of mammals. For this comparison, the reference genomes of domesticated cattle (*Bos taurus*) and its closely related wild species counterpart, bison (*Bison bison*), were used.

**Results:**

It was found that the above-noted highly conserved recombination products (with more than 81.5% identity) were present in the corresponding block of 12 genes in bison. These recombination products served as sources of 51 microRNAs in bison and 129 microRNAs in cattle, including 50 microRNAs that were similar in both species. A total of 79 microRNAs were found only in cattle trinomial recombination products, with 98% belonging to the mir-30 family, including the cattle-specific bta-miR-30a-5p and bta-miR-30e-5p. The mir-30 family is closely associated with biological processes influencing the quantity and quality of agricultural products.

**Conclusion:**

Trinomial retrotransposon recombination products were fixed in both the cattle genome and the genome of its closely related wild species, the bison. It was found that these products may be involved in the response to intensive artificial selection and the domestication process since interspecific differentiation of microRNAs is associated with regulatory networks that have a significant impact on the formation of economically important traits.

## 1 Introduction

*Bos* species (taurine cattle, zebu, yak, river buffalo, swamp buffalo, etc.) have complex patterns of domestication and have been subjects of strong artificial selection (1). The American bison (*Bison bison*) is one of the extant *Bos* species that has not been domesticated (2). Since domesticated and semi-domesticated *Bos* species are well known, cattle and bison represent two extremes on this scale. Therefore, the identification of genomic characteristics that distinguish highly specialized commercial cattle breeds from closely related wild species is of particular interest. However, the genetic factors underlying the domestication of *Bos* species remain unknown (3).

In mammals, mobile genetic elements affect the formation of new genes and their functional evolution. Increased activity of mobile genetic elements can contribute to the formation, subsequent selection, and fixation of new adaptive phenotypic traits during domestication (4, 5). In addition, these elements are capable of forming conserved and variable genomic domains (6–8) with unknown functional features.

Retrotransposons and their recombination products are known to be the main source of new microRNAs, which are an extensive class of single-stranded, short (19–24 bp) non-coding RNAs (9), and they are widely distributed throughout the genome (10–12). Many studies have highlighted the significant importance of microRNAs in the regulation of a wide range of biological processes in different mammalian species (13, 14).

The involvement of microRNA regulatory variants in the selection process plays an important role during domestication and subsequent artificial selection. The origin of modern taurine cattle is closely related to the presence of polymorphic 3′ UTR microRNA binding sites in 1,620 genes of modern-day cattle breeds, compared to its ancestral form, the wild aurochs (*Bos primigenius*). These sites influence neurobiological, metabolic, immunobiological, and reproductive phenotypes associated with domestication (15).

Earlier, we identified 511 domains in bovine chromosome 1 (13,436,028 bp) that were recombination products of the long interspersed nuclear element (LINE) and the endogenous retrovirus (ERV). A total of 30 RTE-BovB/BTLTR1/RTE-BovB clusters (hereinafter BovLTRBov) were found in 12 structural genes (*kcne2, gart, tmem50b, il10rb, ifnar2, urb1, grik1, usp16, ltn1, cyyr1, app*, and *jam2*). These genes form a large syntenic block that has been preserved during the evolution of mammals, starting with the platypus (7, 16). It was found that these BovLTRBov regions are preserved in the bovine genome with high identity, as they are part of its regulatory system containing different microRNAs. Some of these microRNAs are associated with milk and meat production (12).

To determine how these retrotransposon recombination products are involved in the response to intensive artificial selection and, presumably, the domestication process, we compared microRNA-containing homologous regions in cattle and bison. The analysis was conducted using open-source bison genomic sequence data and the sequenced genome of Hereford cattle.

## 2 Results

### 2.1 Bison conservative syntenic group

The functional roles of the 12 structural genes (*kcne2, gart, tmem50b, il10rb, ifnar2, urb1, grik1, usp16, ltn1, cyyr1, app*, and *jam2*) were mostly analyzed in humans (*Homo sapiens*) and laboratory mice (*Mus musculus*), suggesting their close connection with the central nervous system, particularly in relation to the occurrence of behavioral disorders (17, 18), Alzheimer's disease (19–21), and Down syndrome (22). They form an evolutionarily conserved block, which can be found in a number of mammals, including human chromosome 21, mouse chromosome 16, rabbit chromosome 14, and platypus chromosome 17 (16).

**Figure 1:**
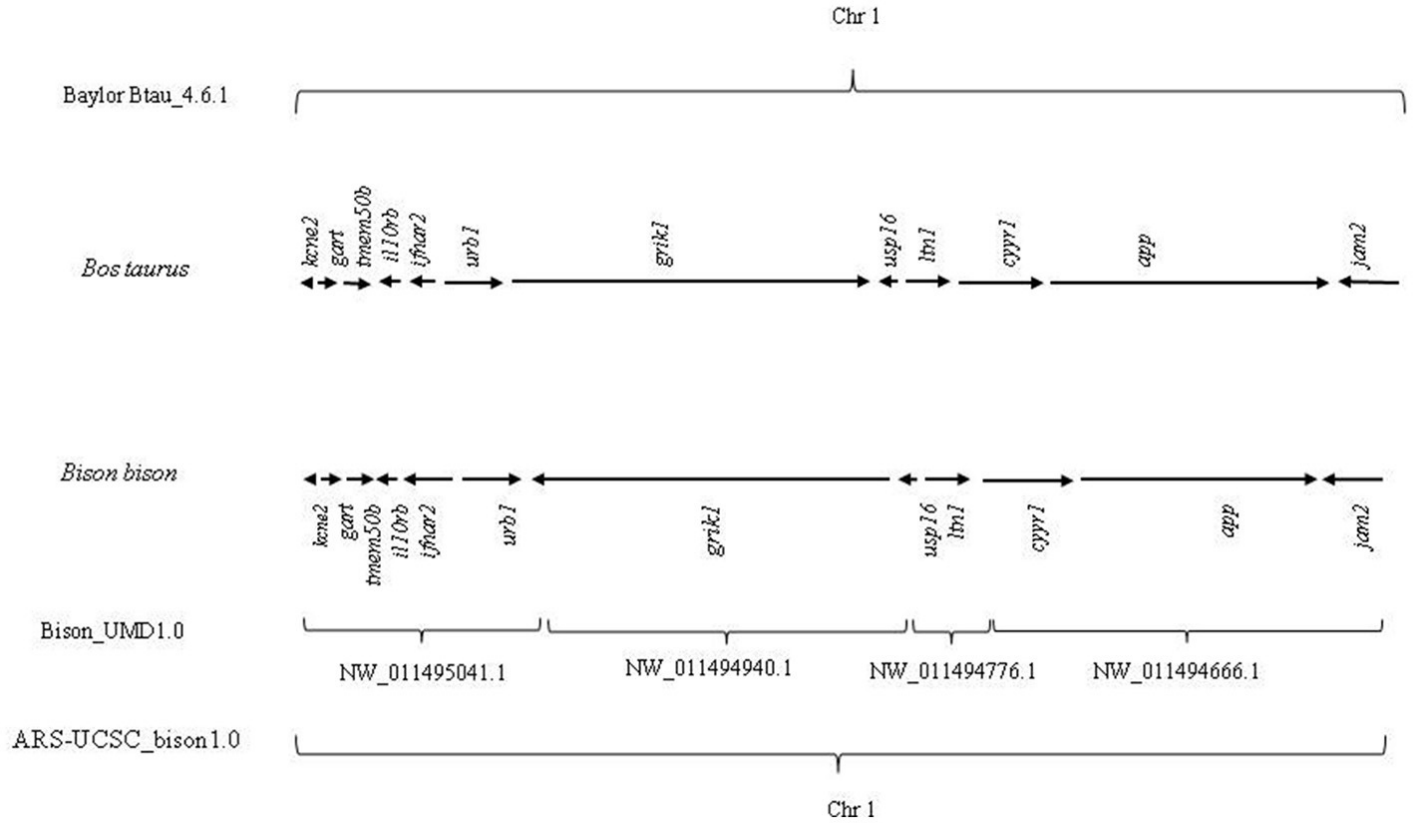
Genomic distribution of evolutionarily conserved 12 genes (*kcne2, gart, tmem50b, il10rb, ifnar2, urb1, grik1, usp16, ltn1, cyyr1, app*, and *jam2*) in *Bos taurus* and *Bison bison*.

They are also present in bison chromosome 1, maintaining the same co-localization (Figure 1).

Pairwise comparisons for 12 genes between bison and cattle demonstrated a high percentage of identity. The maximum percentage of identity was 99.00% (*Bos taurus* phosphoribosylglycinamide formyltransferase, *gart*), and the minimum percentage of identity was 91.00% (*Bos taurus* cysteine and tyrosine rich 1, *cyyr1*) (Table 1).

**Table 1:** Genomic characteristics for 12 evolutionarily conserved genes (*kcne2, gart, tmem50b, il10rb, ifnar2, urb1, grik1, usp16, ltn1, cyyr1, app, jam2*) in cattle (*ver*. 2011) and bison (*ver*. 2014).

**No**	**Gene**	**%**	***Bos taurus*** **(cattle)**					***Bison bison***					
			**Chr**	**Beginning**	**End**	**Length**	**Orientation**	**Chr**	**Location**	**Beginning**	**End**	**Length**	**Orientation**
1	*kcene2*	96.70	1	460,824	468,632	7,809	Complement	Un	NW_011495041.1	438,216	446,126	7,911	Complement
2	*gart*	99.00	1	1,064,795	1,091,084	26,290		Un	NW_011495041.1	1,304,517	1,331,783	27,267	
3	*tmem50b*	97.70	1	1,125,432	1,162,271	36,840		Un	NW_011495041.1	1,366,487	1,403,341	36,855	
4	*il10rb*	97.80	1	1,364,073	1,392,682	28,610	Complement	Un	NW_011495041.1	1,605,601	1,634,436	28,836	Complement
5	*ifnar2*	97.60	1	1,394,236	1,428,725	34,490	Complement	Un	NW_011495041.1	1,635,891	1,701,341	65,451	Complement
6	*urb1*	94.50	1	2,181,487	2,258,468	76,982		Un	NW_011495041.1	2,425,279	2,503,498	78,220	
7	*grik1*	94.00	1	5,245,035	5,717,636	472,602		Un	NW_011494940.1	3,737,498	4,211,412	473,915	Complement
8	*usp16*	98.40	1	6,365,264	6,391,124	25,861	Complement	Un	NW_011494776.1	270,105	296,092	25,988	Complement
9	*ltn1*	97.20	1	6,419,776	6,476,700	56,925		Un	NW_011494776.1	326,250	385,753	59,504	
10	*cyyr1*	91.00	1	9,186,471	9,301,517	115,047		Un	NW_011494666.1	1,283,391	1,406,400	123,010	
11	*app*	95.00	1	9,540,541	9,909,501	368,961		Un	NW_011494666.1	1,653,242	1,966,900	313,659	
12	*jam2*	94.60	1	10,014,214	10,096,812	82,599	Complement	Un	NW_011494666.1	2,127,848	2,211,323	83,476	Complement

Furthermore, intron-located regions of bovine chromosome 1, homologous to BovLTRBov, were also found in bison for these genes. The maximum percentage of identity was 99.63% (*Bos taurus* phosphoribosylglycinamide formyltransferase, *gart*), and the minimum was 81.5% (*Bos taurus* junctional adhesion molecule 2, *jam2*) (Table 2). The frequency of BovLTRBov was 32% higher in cattle than in bison.

**Table 2:** Percent identity matrix of the RTE-BovB/BTLTR1/RTE-BovB recombination products in cattle and bison (pairwise alignment) (%).

**No**.	**Cluster**	**%**	**No**.	**Cluster**	**%**	**No**.	**Cluster**	**%**
**1**	**2**	**3**	**1**	**2**	**3**	**1**	**2**	**3**
1	+1-*gart*	99.63	11	+11-*grik1*	98.0	21	c1-*kcne2*	93.49
2	+2-*tmem50b*	99.07	12	+12-*grik1*	96.9	22	c2-*grik1*	97.9
3	+3-*il10rb*	98.84	13	+13-*ltn1*	87.03	23	c3-*grik1*	91.0
4	+4-*il10rb*	99.07	14	+14-*app-*	91.7	24	c4-*grik1*	93.3
5	+5-*ifnar2*	99.47	15	+15-*app*	97.7	25	c5-*usp16*	97.99
6	+6-*urb1*	98.00	16	+16-*app*	99.0	26	c6-*cyyr1*	98.51
7	+7-*grik1*	98.6	17	+17-*app*	89.2	27	c7-*app*	89.2
8	+8-*grik1*	98.1	18	+18-*app*	84.9	28	c8-*app*	87.8
9	+9-*grik1*	97.7	19	+19-*app*	84.8	29	c9-*app*	84.9
10	+10-*grik1*	98.0	20	+20-*jam2*	81.5	30	c10-*jam2*	99.12

It was found that regions that closely match six bovine BovLTRBov recombination products in *Bos taurus* amyloid beta precursor protein, *app*, were all present in the same bison sequence of 2,772 bp in length, which is also part of the *app* (the coordinates are shown in Table 3).

**Table 3:** Bison sequence coordinates (within the corresponding genes) with a high percentage of identity to the RTE-BovB/BTLTR1/RTE-BovB recombination products in the cattle.

**No**.	**Cluster**	**Beginning**	**End**	**Length**	**No**.	**Cluster**	**Beginning**	**End**	**Length**
1	1- *gart*-Bis	6,481	8,348	1,868	16	C5- *usp16*-Bis	23,727	24,125	399
2	2- *tmem50b*-Bis	22,859	25,875	3,017	17	C6- *cyyr1*-Bis	27,214	29,161	1,948
3	3- *il10rb*-Bis	24,068	25,872	1,805	18	C7- *app*-Bis	238,752	241,966	3,215
4	4- *il10rb*-Bis	15,573	16,759	1,187	19	C3- *grik1*-Bis	286,114	287,632	1,519
5	5- *ifnar2*-Bis	43,219	44,162	944		9- *grik1*-Bis	286,116	288,060	1,945
6	6- *urb1*-Bis	51,345	54,346	3,002	20	10- *grik1*-Bis	359,675	361,233	1,559
7	7- *grik1*-Bis	147,651	150,567	2,917		11- *grik1*-Bis	359,675	361,233	1,559
8	8- *grik1*-Bis	215,970	216,535	566	21	C8- *app*-Bis	164,412	166,018	1,607
9	12- *grik1*-Bis	466,917	468,534	1,618		C9- *app*-Bis	164,769	165,870	1,102
10	13- *ltn1*-Bis	40,839	42,607	1,769		15- *app*-Bis	164,433	167,183	2,751
11	14- *app*-Bis	24,343	27,080	2,738		17- *app*-Bis	164,433	166,087	1,655
12	16- *app*-Bis	205,433	206,385	953		18- *app*-Bis	164,430	166,591	2,162
13	C1- *kcne2*-Bis	1,569	3,525	1,957		19- *app*-Bis	164,433	166,715	2,283
14	C2- *grik1*-Bis	110,695	113,631	2,937	22	C10- *jam2*-Bis	75,734	78,221	2,488
15	C4- *grik1*-Bis	276,452	278,785	2,334		20- *jam2*-Bis	76,649	78,816	2,168

One sequence from *Bos taurus* junctional adhesion molecule 2, *jam 2*, which is 3,083 bp long in bison, showed a high degree of homology to two bovine BovLTRBov regions located within the same gene (Table 3).

Two pairs of highly homologous bovine BovLTRBov regions from *Bos taurus* glutamate ionotropic receptor kainate type subunit 1, *grik1*, were found in two sections of the bison *grik1* gene, measuring 1,559 bp and 1,947 bp in length (Table 3).

**Figure 2:**
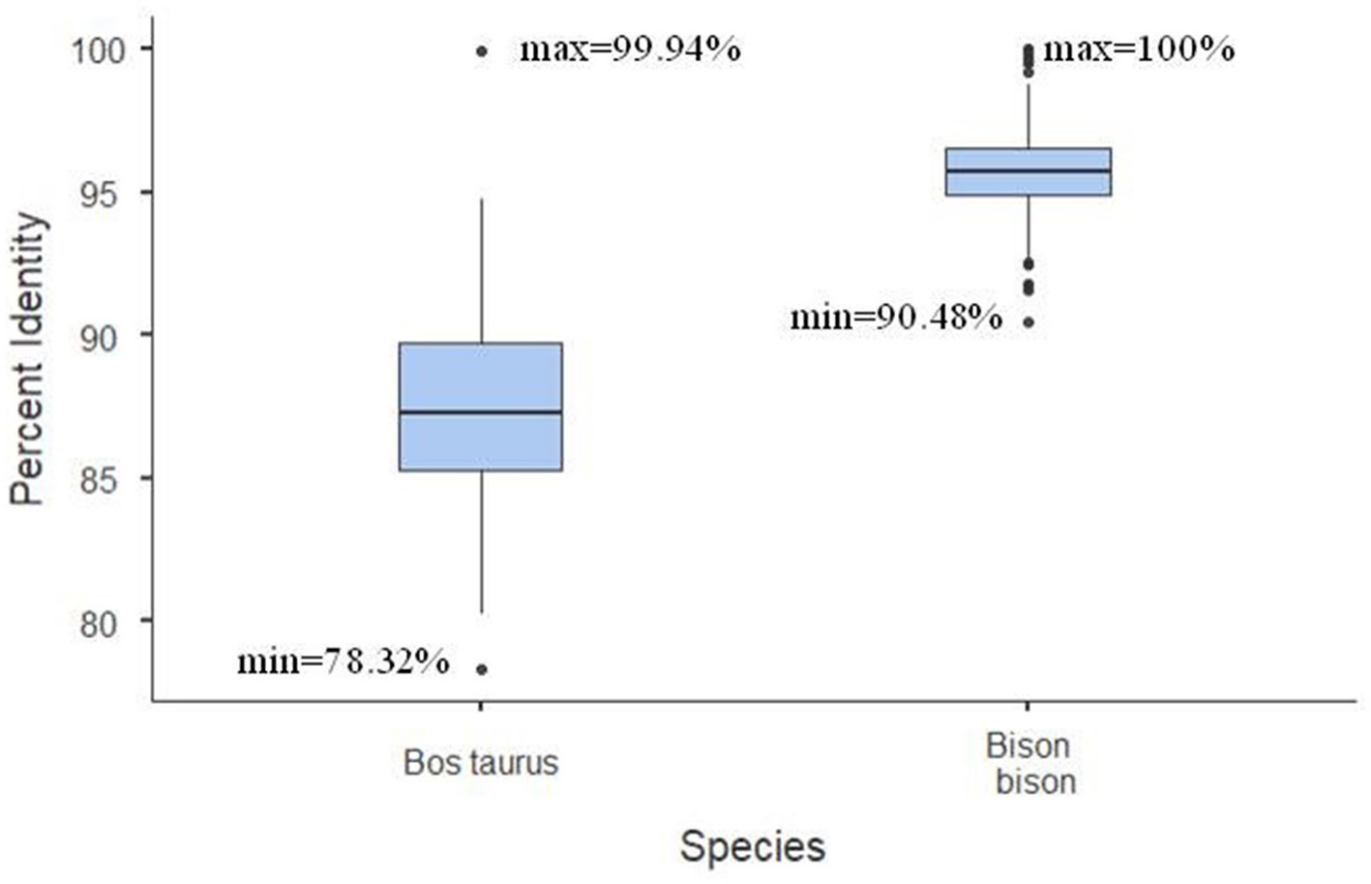
Percent identity matrix. The heatmap shows the bison sequences with a high percentage of identity to RTE-BovB/BTLTR1/RTE-BovB recombination products in cattle (multiple sequence alignment). The colors indicate the percent identity between the sequences. Green represents 90% identity, red represents 100% identity, and other colors represent values in between.

Multiple sequence alignments of all bison sequences with a high percentage of identity to bovine BovLTRBov showed a minimum percent identity of 90.48% (sequences in listerin E3 ubiquitin protein ligase 1, *ltn1*, and potassium voltage-gated channel subfamily E regulatory subunit 2, *kcne2*) and a maximum of 100% (between two sequences in amyloid beta precursor protein, *app*) (Figure 2).

**Figure 3:**
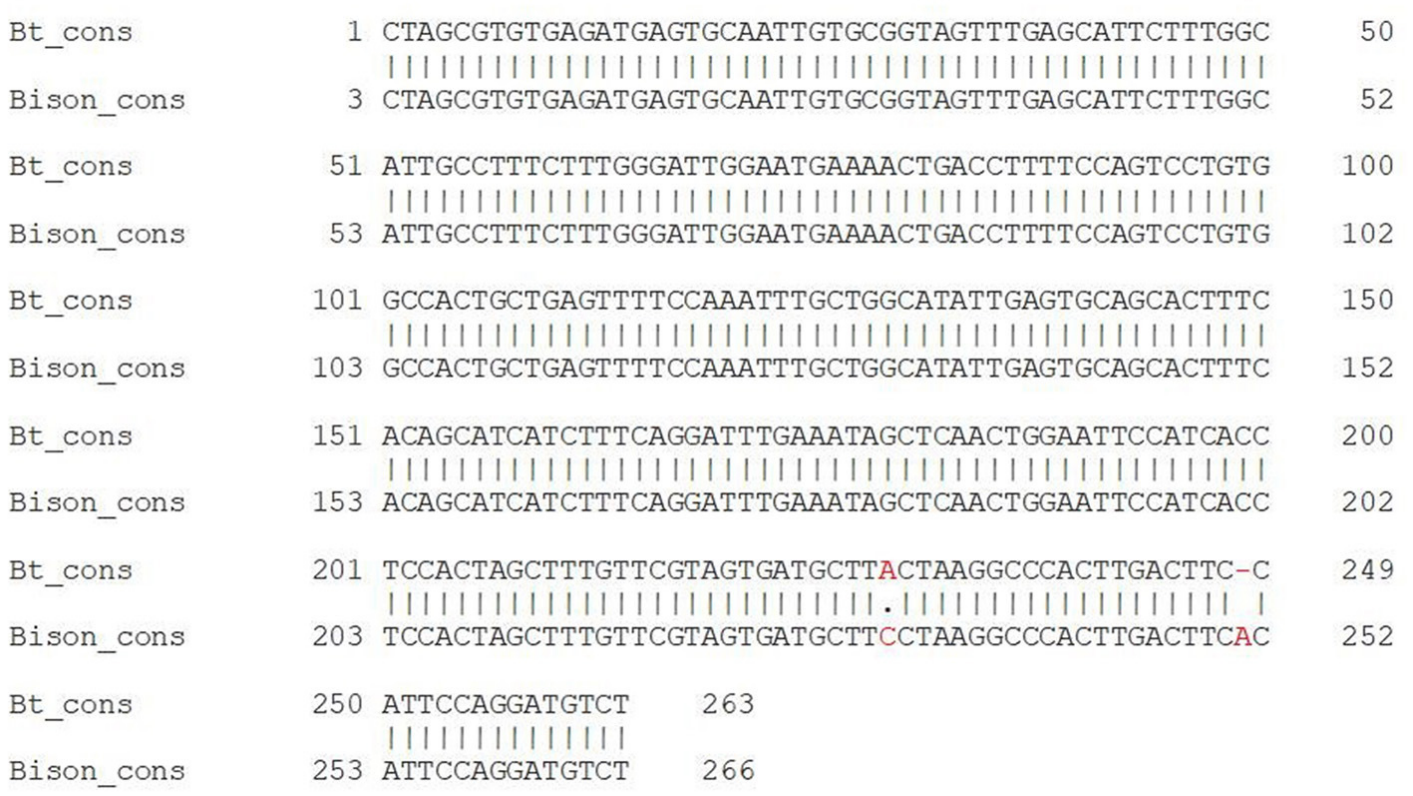
The percent identity between all the detected regions in the gene block in the *Bos taurus* and *Bison bison*.

The percent identity of these bison regions was higher than that of the cattle recombination products (Figure 3).

The high similarity between BovLTRBov regions within the genes from the studied wild-domesticated pair suggested their specific functional roles. This strong conservation may be due to the presence of regulatory network elements such as microRNAs. The regions, homologous to microRNAs, were further searched in the cattle and bison to test this assumption.

### 2.2 Analysis of microRNA presence

The following sequence (266 bp) was identified from the Bison regions homologous to bovine 30 BovLTRBov, with at least 95.29% similarity (Table 4):

**Table 4:** Percent identity matrix of the bison conserved sequence (CS) with the identified nucleotide sequences (%).

**Cluster**	**1- *gart*-Bis_c**	**2-tmem50b-Bis_c**	**3-*il10rb*-Bis_c**	**4-*il10rb*-Bis-c**	**5-*ifnar2*-Bis**	**6-*urb1*-Bis_c**	**7- *grik1*-Bis_c**	**8- *grik1*-Bis_c**	**9- *grik1*-Bis**	**10- *grik1*-Bis**
Cons	95.29	96.18	97.33	95.6	99.62	97.33	98.46	98.07	98.07	99.23
**Cluster**	**11-** ***grik1*****-Bis**	**12-** ***grik1*****-Bis**	**13-** ***ltn1*** **-Bis**	**14-** ***app*****-Bis**	**15-** ***app*****-Bis**	**16-** ***app*****-Bis**	**17-** ***app*****-Bis_c**	**18-** ***app*****-Bis**	**19-** ***app*****-Bis**	**20-** ***jam2*****-Bis**
Cons	99.23	97.35	98.8	96.43	98.48	98.48	97.7	98.48	98.48	97.3
**Cluster**	**c1-KCNE2-Bis_c**	**C2-** ***grik1*****-Bis_c**	**C3-** ***grik1*****-Bis_c**	**C4-** ***grik1*****-Bis_c**	**C5-** ***usp16*****-Bis**	**C6-** ***cyyr1*****-Bis_c**	**C7-** ***app*****-Bis**	**C8-** ***app*****-Bis**	**C9-** ***app*****-Bis**	**C10-** ***jam2*****-Bis**
Cons	98.05	98.05	97.67	97.95	99	97.34	98.48	96.6	98.48	97.31

TACTAGCGTGTGAGATGAGTGCAATTGTGC GGTAGTTTGAGCATTCTTTGGCATTGCCTTTC TTTGGGATTGGAATGAAAACTGACCTTTTCCAGTCCTGT GGCCACTGCTGAGTTTTCCAAATTTGCTGG CATATTGAGTGCAGCACTTTCACAGCATCAT CTTTCAGGATTTGAAATAGCTCAACTGG AATTCCATCACCTCCACTAGCTTTGTTC GTAGTGATGCTTCCTAAGGCCCACTTGACTTCACATTCCAGGATGTCT.

**Figure 4:**
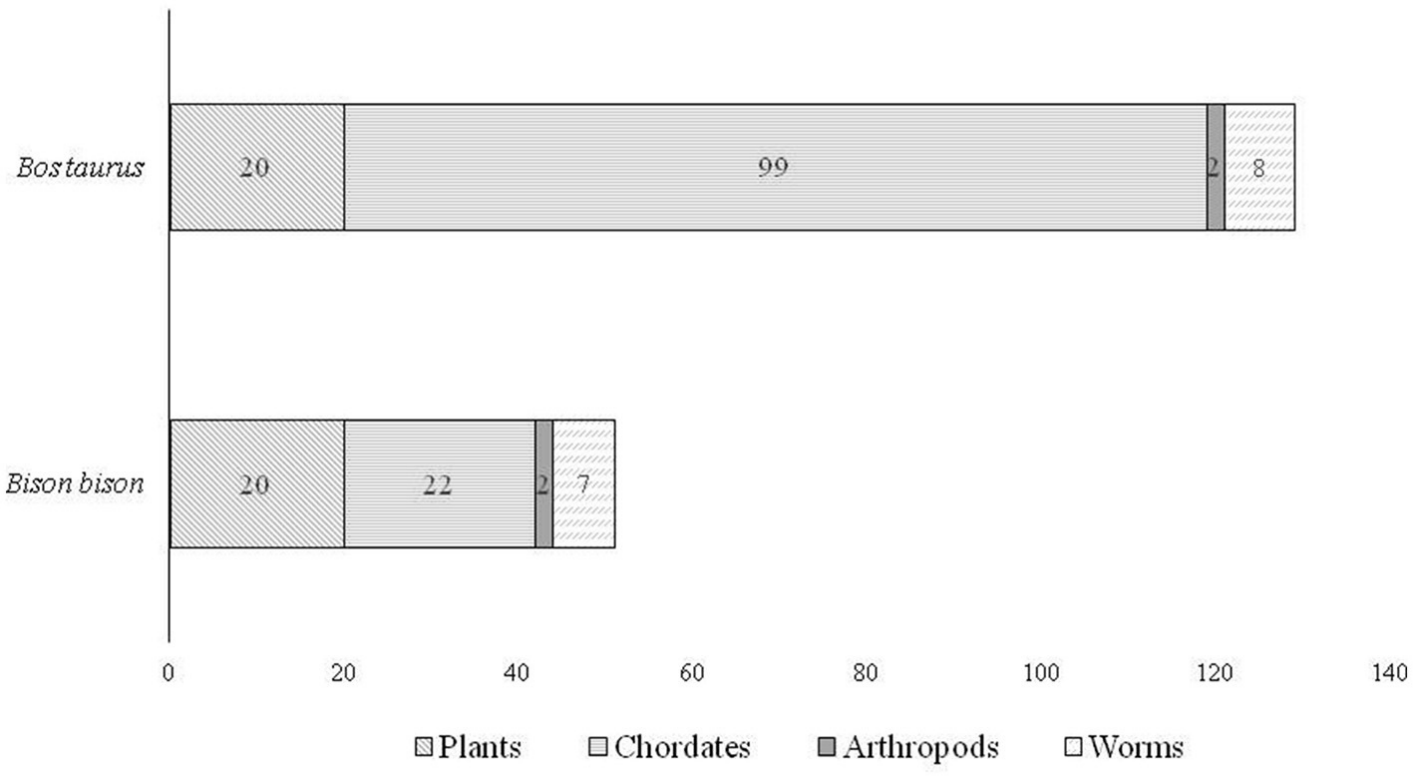
Alignment of the bison (*Bison bison)* conserved sequence with the cattle conserved sequence, as reported by Skobel et al. (12).

Earlier, we identified a conserved sequence of 266 bp in length from recombination products in cattle (12). The percent identity between the conserved sequences of cattle and bison was 99.2% (Figure 4).

**Figure 5:**
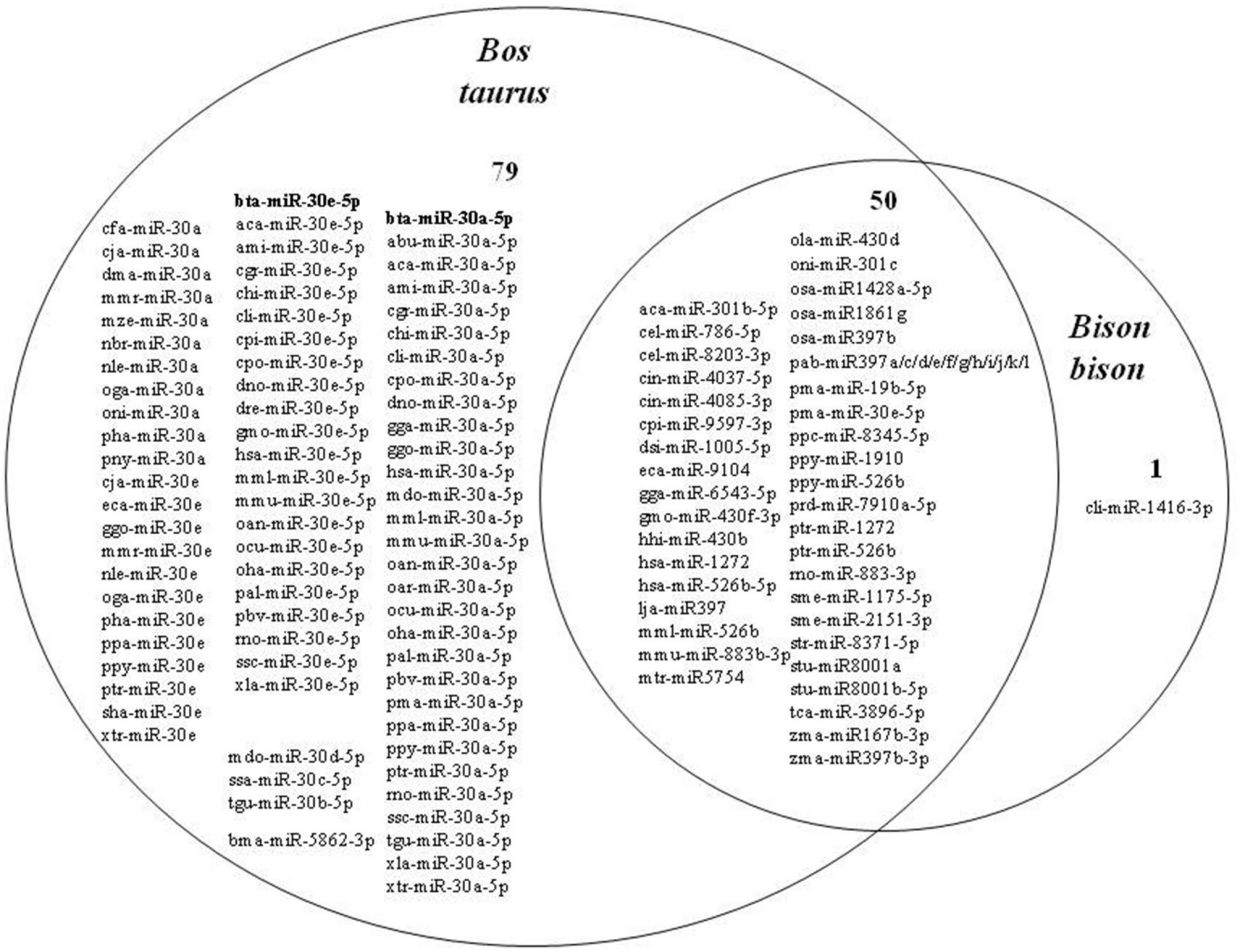
Distribution of microRNAs present in the conserved sequences in bison (*Bison bison* and *Bos taurus)*.

The search for microRNAs in the conserved bison sequence resulted in 51 microRNAs from 30 different species (both animals and plants). A total of 129 microRNAs from 63 different species were found in the *Bos taurus* conserved sequence (Figure 5).

**Figure 6:**
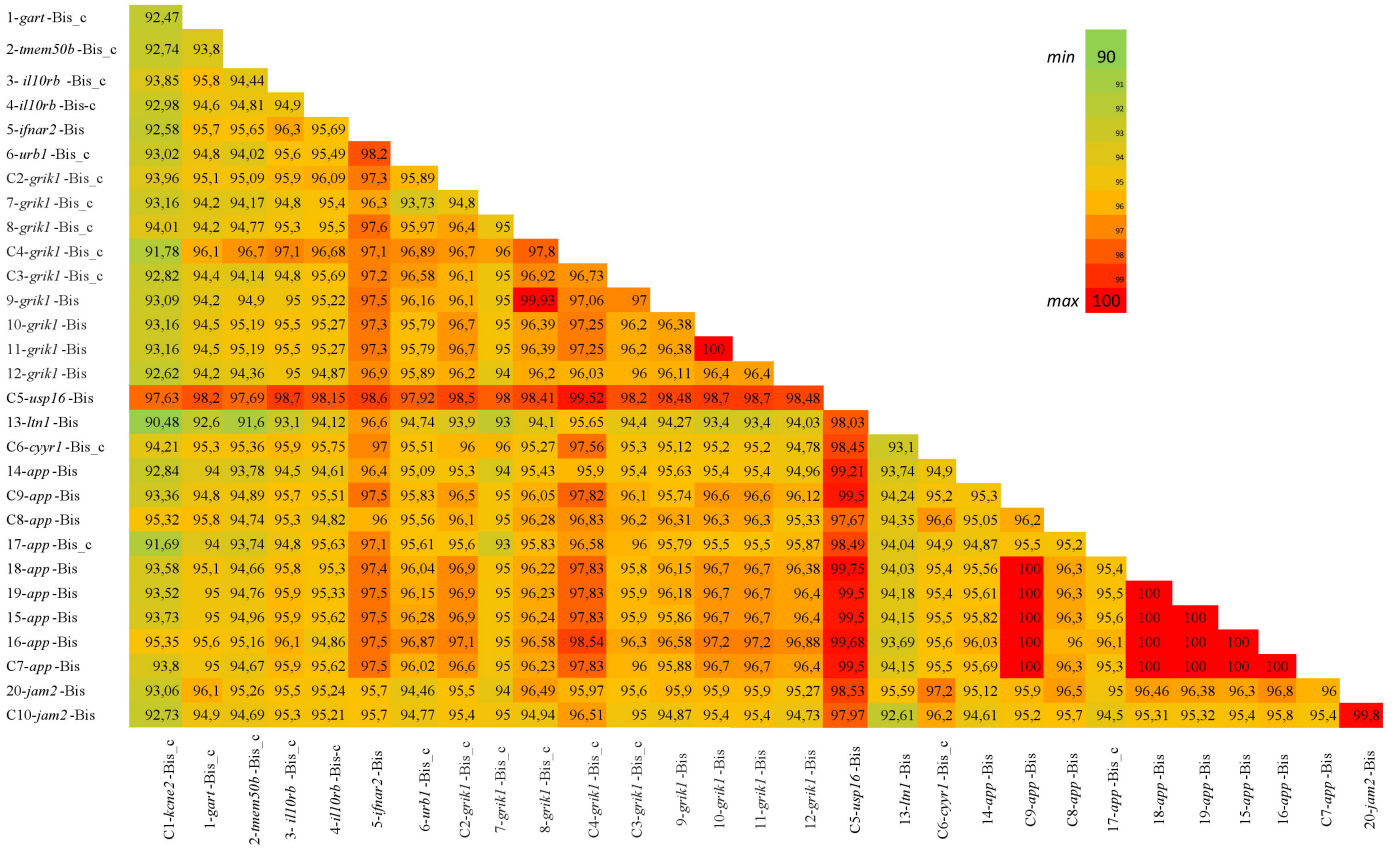
Comparison of microRNAs found in the *Bos taurus* and *Bison bison* syntenic gene block.

A total of 50 microRNAs were identified as common between cattle and bison (Figure 6). For example, mtr-miR-5754 *(Medicago truncatula*, barrel clover*)*, found in the conserved sequence of both bison and cattle, is known to decrease the stability of oncogenic target transcripts in humans, whose products promote cell proliferation (23).

The most similar plant-specific microRNAs (14 out of 20) belonged to the miR-397 family. MiR-397 is involved in various biological processes, including cell growth, reproductive organ development, and plant resistance to external adverse stimulation. Additionally, it also is involved in regulating gene functions related to fatty acid metabolism (24).

MiR-526b overexpression is statistically significant in patients with bipolar disorder (25). MiR-526b is also associated with various types of oncological diseases, such as cervical cancer (26) and breast cancer (27). Moreover, MiR-1272 plays a crucial role in the regulation of immune signaling, cytokine production, and migration of immune cells in order to control visceral leishmaniasis infection in humans (28). Eca-miR-9104 is expressed in horses during equine herpesvirus 1 infection (29). However, for the majority of microRNAs homologous between cattle and bison, their functional roles remain to be studied.

At the same time, it was found that one microRNA, cli-miR-1416-3p, was absent in cattle but present in bison. This microRNA belongs to the miR-1416-3p family and is presumably involved in follicle atresia during specific stages of folliculogenesis in birds (30).

A total of 79 microRNAs were absent in bison but present in cattle, including 78 microRNAs belonging to the miR-30 family, with 2 bovine-specific microRNAs, namely bta-miR-30a-5p and bta-miR-30e-5p.

The accumulated evidence suggests that miR-30a-5p and miR-30e-5p are crucial for regulating key physiological systems in cattle. They play a key role in the response to heat stress (31), affect the development of the immune system and the immune response (32), are vital for milk production (85, 86), can influence milk composition (81, 82), inhibit the differentiation of muscle cells (33), and play an important regulatory role in the processes of fertilization and early development (34).

MiR-30e-5p and miR-30a-5p are also involved in the process of aging in humans (35) and play a role in the regulation of various diseases, including bacterial infections (36). They are considered potential biomarkers for neurodegenerative disorders (37), systemic lupus erythematosus (38), diabetes (39), various tumors (40), and heart diseases (83, 84).

## 3 Discussion

It is becoming clear that domesticated species differ from their closely related wild counterparts in terms of a high level of phenotypic variability (41). Understanding the evolution of the genetic mechanisms behind phenotypic traits has become possible by comparing closely related species with their wild counterparts and identifying key elements that regulate underlying variability (42).

Nonetheless, selecting the appropriate wild-domesticated pair has become a fundamental consideration. Along with the European bison (*Bison bonasus*) and the gaur (*Bos gaurus*), the American bison is one of the few remaining wild animals of the genus *Bos* (2). The gaur, on the other hand, has a domesticated form known as the gayal, and it has been noted that artificial selection has an impact on the gaur genome (43). In contrast, no domesticated form of bison is known, and evidence suggests that bovine alleles may only be present in a limited portion of the genome (44).

To date, a large amount of public data on the organization of the bovine genome has been accumulated (45–47). We used open-source Hereford cattle (*Bos taurus*) gene data based on the Baylor Btau_4.6.1/bosTau7 October 2011 assembly to maintain the logic and reproducibility of our previous research. It should be noted that for the purpose of this study, the use of the latest version of the cattle reference genome [The ARS-UCD1.3 (47)] did not affect the results as the sequences of the studied structural genes exhibited high similarity across different versions (Supplementary material). Modern sequencing technologies generate new high-quality assemblies, reducing possible errors in sequences enriched with elements such as CG repeats (48, 49). However, the type of animal used for genome obtaining is also of great importance. The reference scaffold-level bison genome, Bison_UMD1.0, is currently available in the NCBI database (50). A more recent genome assembly, ARS-UCSC_bison1.0, (51) exists, but this genome represents an F1 hybrid between a bison sire and a Simmental cow. To avoid potential hybridization effects (52, 53), we excluded this assembly from the analysis.

In the majority of cases of speciation, genetic information cannot be traced through the evolution of particular genes but only through gene clusters (54). Synteny analysis, that is, studying the conserved blocks of genes found in different species, is one of the comparative genomics methods used for understanding evolutionary relationships, including those during domestication (55–57). Genomic studies typically rely on closely linked and conserved gene clusters and also require an understanding of the functional features of the genes included in these clusters (58, 59).

The 12 genes mentioned in this study form a large syntenic block, maintained during the evolution of mammals, and are notable for their well-conserved synteny (7, 12). These genes are involved in social activity, which is a crucial component of the domestication process (60). Our results also support the accumulated evidence that evolutionarily conserved syntenic blocks have a higher density of genes involved in the formation of anatomical characteristics and the development of the central nervous system (61). Hence, these genes are also believed to be associated with animal socialization and, therefore, domestication (62, 63). *Gart, il10rb, ifnar2, urb1*, and *ltn1* are also considered candidates for bovine artificial selection (64–66). In cattle, *tmem50B* and *app* are candidate domestication genes according to the presence of divergent microRNA binding sites (15).

The pairwise comparison of the full-length genes indicated they are highly conserved in both *Bos* genus species. Therefore, the identified differences in the presence and genomic position of the microRNAs, which are involved in the regulatory networks of gene expression, are intriguing.

The Bison regions, homologous to bovine BovLTRBov, were never found in exons of either bison or cattle. This may be because the consensus sequence is too long to be present in exons without interfering with the genes' functions. In addition, they had a lower frequency but higher pairwise identity compared to cattle. It probably reflects the differences between the two species in phenotypic variability and the width of species distribution (1). In addition, the higher proportion of retrotransposons in domesticated animals compared to their wild counterparts is likely related to the domestication process (67).

Retrotransposons and their genome distribution are very species-specific. For example, LINE/RTE-BovB is frequently found only in *Bos* species, despite being involved in horizontal genetic information transfer (68). This situation is similar to the presence of SINE/Alu in primate genomes and SINE/tRNA-Core-RTE in cattle genomes. Even very different transposons, such as the LINE, SINE, and long terminal repeat (LTR), can sometimes contain the same regulatory elements (69).

The *in silico* microRNA identification strategy is used as an accurate, fast, and reliable method for predicting microRNA homologs in different species (70, 71). The proportion of microRNAs derived from transposons in humans is higher than in other vertebrates, especially non-mammal vertebrates (72). We provide evidence that the number of microRNAs derived from transposons also increases during the domestication process (Figure 6). It is important to note that multiple hybridization events between *Bos taurus* and *Bison bison* have taken place over the last 200 years (44). Nevertheless, our study revealed interspecific differences in microRNA presence. Considering the high identity of the two conserved sequences, a number of microRNAs were found in both cattle and bison. The likely diversity of species-specific microRNAs has been proven by existing scientific data. Recent evidence suggests that microRNAs can move from plants to animals via the gastrointestinal tract and access cellular targets, affecting the physiological and pathological conditions of their recipients (73).

The most interesting result of our study is that some microRNAs were different between the genomes of cattle and bison. This occurred because of the variations in the conserved sequences of the two species in three nucleotides at the beginning and the presence of a single-nucleotide polymorphism (SNP) in the bison conserved sequence at 251 bp (Figure 4). Of these, 78 microRNAs were from the miR-30 family, including two bovine-specific microRNAs: bta-miR-30a-5p and bta-miR-30e-5p. Members of this family are known to increase milk fat content (74), contribute to the development of muscle tissue in cattle (75), and play a role in the development of stress and immune responses (32). Since the microRNAs that differed between the two species were related to important agricultural differences between cattle and bison, we assumed that these differences result from intensive artificial selection.

In general, the obtained results indicate that the accumulation of retrotransposons and their recombination products may be a source of microRNA regulatory networks. Our comparative analysis of the LINE and ERV sequences in a domesticated species (*Bos taurus*) and a closely related wild species (*Bison bison*) at 12 loci, where synteny has been maintained since the early stages of evolution, suggests the identification of molecular genetic pathways underlying the response to intensive artificial selection and, presumably, the domestication of *Bov* species. It should be noted that these gene products are likely to be involved in higher central nervous system activity in mammals.

Frequently, genome assemblies are insufficient for comparative genomic analysis as they may limit further interpretation by failing to capture the entire range of genetic diversity within a species (76, 77). However, in our study, we analyzed the sequences located in structural genes that preserve genetic linkage during evolution in different species. The clear difference we found in microRNA presence between the cattle and bison and the existing data on the formation of new binding sites during bovine domestication (15) both support our hypothesis that microRNAs could be involved in the process of artificial selection.

## 4 Materials and methods

The bison annotation release (IDs: 237421 [UID] 1351428 [GenBank] 1426508 [RefSeq]) (50) was retrieved from the NCBI GenBank (GenBank, RRID:SCR_002760). We used the Bison chromosome-scale genome, ARS-UCSC_bison1.0, to identify gene locations (51).

All gene data of Hereford cattle (*Bos taurus*) were based on the Baylor Btau_4.6.1/bosTau7 October 2011 assembly available in the Integrated Genome Browser (IGB, RRID:SCR_011792) to maintain the logic and reproducibility of our previous research. The ARS-UCD1.3 reference bovine genome was used to confirm the reliability and accuracy of the sequences utilized (47). The results are presented in Supplementary material.

The available cow RepeatMasker genomic dataset was used for obtaining information on the distribution of mobile genetic elements and their positioning in the cattle genome (78, 79). We identified 511 trinomial recombination products RTE-BovB/BTLTR1/RTE-BovB between the endogenous retrovirus (ERV) containing the LTR BTLTR1 and the non-LTR long interspersed nuclear element (LINE) RTE-BovB in the 13,436,028 bp nucleotide sequences of bovine chromosome 1. For further analysis, we took 30 RTE-BovB/BTLTR1/RTE-BovB (hereinafter BovLTRBov) recombination products detected in the 12 structural genes (*kcne2, gart, tmem50b, il10rb, ifnar2, urb1, grik1, usp16, ltn1, cyyr1, app, and jam2*), while the rest were found in intergenic spaces (7).

The coordinates of the 30 cattle RTE-BovB/BTLTR1/RTE-BovB recombination products according to the Baylor Btau_4.6.1/bosTau7 October 2011 assembly are indicated in Supplementary material.

Our previous studies (7, 12) have provided detailed methods for detecting trinomial recombination products between the LINE and LTR ERV, analyzing their localization in relation to structural genes and identifying the RTE-BovB/BTLTR1/RTE-BovB conserved sequence.

Open-source software provided by the European Institute of Bioinformatics was used to identify regions of similarity between the nucleotide sequences of cattle (*Bos taurus*) and bison (*Bison bison*). We used Clustal Omega (Clustal Omega, RRID:SCR_001591) and Kalign (Kalign, RRID:SCR_011810) for multiple sequence alignments with default settings. The EMBOSS Matcher (EMBOSSMatcher, RRID:SCR_017252) option during the pairwise sequence alignment was changed. We set the maximum value of alternative matches to 20 to ensure that possible additional alignments were not missed, while keeping all other parameters at their default settings.

The conserved sequence from the bison regions homologous to bovine was identified manually based on the results obtained from Kalign (Kalign, RRID:SCR_011810).

To check the presence of microRNAs in the conserved sequences of both bison and cattle, we used the microRNA database (v20), sorted by E-value, with the maximum possible number of results set to be displayed (80).

## 5 Conclusion

It can be assumed that the trinomial recombination products between the LINE and the ERV are fixed in the genomes of cattle and the closely related wild species, bison. These products could be actively involved in the response to intensive artificial selection and the domestication process by serving as sources of microRNAs that have a significant impact on agriculturally important cattle traits. Consequently, regulatory networks could change significantly under intensive artificial selection and probably domestication, not only due to the origin of new microRNA binding sites (15) but also due to the formation of new microRNAs.

Future studies are needed to validate these results by examining other wild-domesticated pairs of vertebrates and verifying the functional association with the observed differentiation of microRNA.

## Data Availability

The original contributions presented in the study are included in the article/Supplementary material, further inquiries can be directed to the corresponding author.
