# Supplementary material for: Bos taurus and Bison bison conservative retrotransposon recombination products
Source: Front Vet Sci. 2025 Apr 30;12:1516731. doi: 10.3389/fvets.2025.1516731 (PMC12075945; doi:10.3389/fvets.2025.1516731)
Supplement: Supplementary file 1 [file Table_1.doc]

**Supplementary Materials.**

**1.** **The comparison of the full-length sequences of 12 genes (*kcne2, gart, tmem50b, il10rb, ifnar2, urb1, grik1, usp16, ltn1, cyyr1, app, jam2*) taken from the Baylor versions Btau_4.6.1/bosTau7 October 2011 and ARS-UCD1.3.**

Realizing the importance of actual genome versions studying, we compared the full-length sequences of 12 genes (*kcne2, gart, tmem50b, il10rb, ifnar2, urb1, grik1, usp16, ltn1, cyyr1, app, jam2*) taken from the Baylor versions Btau_4.6.1/bosTau7 October 2011 and ARS-UCD1.3 with EMBOSS Matcher. Their percentage of homology turned out to be not less than 97.9% (*grik1*, with the longest sequences in 472602 bp and 466871 bp, respectively) (Table 1)

**Table 1. Pairwise sequence comparison of 12 gene taken from Baylor Btau_4.6.1/bosTau7 October 2011(2011) and ARS-UCD1.3** (2018)

| **№** | **Gene** | **%** | **Length** | | |
| --- | --- | --- | --- | --- | --- |
|  |  |  | **2011** | **2018** | **Odds** |
| 1 | *kcne2* | 100 | 7809 | 7808 | 1 |
| 2 | *gart* | 100 | 26290 | 26291 | 1 |
| 3 | *tmem50b* | 100 | 36840 | 36842 | 2 |
| 4 | *il10rb* | 99,4 | 28610 | 28670 | 60 |
| 5 | *ifnar2* | 100 | 34490 | 34490 | 0 |
| 6 | *urb1* | 99,6 | 76982 | 77078 | 96 |
| 7 | *grik1* | 97,9 | 472602 | 466871 | 5731 |
| 8 | *usp16* | 100 | 25861 | 25935 | 74 |
| 9 | *ltn1* | 99,9 | 56925 | 58783 | 1858 |
| 10 | *cyyr1* | 99,6 | 115047 | 216118 | 101071 |
| 11 | *app* | 98,7 | 368961 | 312523 | 56438 |
| 12 | *jam2* | 99,8 | 82599 | 82562 | 37 |

We also verified the presence of the 30 studied RTE-BovB/BTLTR1/RTE-BovB recombination products in the corresponding structural genes from the ARS-UCD1.2 version. They show strong conservatism too (Table 2).

**Table 2. The presence of 30 RTE-BovB/BTLTR1/RTE-BovB in the corresponding structural genes from the ARS-UCD1.3 version**

| **№** | **Cluster** | **%** |  | **№** | **Cluster** | **%** |  | **№** | **Cluster** | **%** |
| --- | --- | --- | --- | --- | --- | --- | --- | --- | --- | --- |
| **1** | **2** | **3** |  | **1** | **2** | **3** |  | **1** | **2** | **5** |
| 1 | 1-gart-Bt | 100 |  | 11 | 11-grik1-Bt | 99 |  | 21 | C1-kcne2-Bt | 100 |
| 2 | 2-tmem50b-Bt | 100 |  | 12 | 12-grik1-Bt | 98,6 |  | 22 | C2-grik1-Bt | 100 |
| 3 | 3-il10rb-Bt | 97,9 |  | 13 | 13-ltn1-Bt | 99,9 |  | 23 | C3-grik1-Bt | 100 |
| 4 | 4-il10rb-Bt | 99,9 |  | 14 | 14-app-Bt | 100 |  | 24 | C4-grik1-Bt | 99,9 |
| 5 | 5-ifnar2-Bt | 100 |  | 15 | 15-app-Bt | 99,6 |  | 25 | C5-usp16-Bt | 100 |
| 6 | 6-urb1-Bt | 100 |  | 16 | 16-app-Bt | 100 |  | 26 | C6-cyyr1-Bt | 100 |
| 7 | 7-grik1-Bt | 99,9 |  | 17 | 17-app-Bt | 99,9 |  | 27 | C7-app-Bt | 100 |
| 8 | 8-grik1-Bt | 98,8 |  | 18 | 18-app-Bt | 89,4 |  | 28 | C8-app-Bt | 100 |
| 9 | 9-grik1-Bt | 99,9 |  | 19 | 19-app-Bt | 87,4 |  | 29 | C9-app-Bt | 88,1 |
| 10 | 10-grik1-Bt | 98,9 |  | 20 | 20-jam2-Bt | 100 |  | 30 | C10-jam2-Bt | 100 |

Of particular interest are the sequences that have a minimal percentage of homology with the recombination products. They are 18-app-Bt (89.4%), 19-app-Bt (87.4%), and C9-app-Bt (88.1%). It should be noted that the *app* sequence in the Baylor Btau_4.6.1/bosTau7 October 2011 reference genome is 56438 bp longer than that in the ARS-UCD1.3 reference genome (Table 1). These three recombination products are located at the distal end of *app* from the Baylor Btau_4.6.1/bosTau7 October 2011 which is absent in ARS-UCD1.3 (Table 3). They are not unique and can be excluded from the analysis in case of ARS-UCD1.3. use.

**Table 3. The coordinate analysis of the *app* ARS-UCD1.3 gene sequences that have a high percentage of homology with the 9 RTE-BovB/BTLTR1/RTE-BovB sequences ( ver. 2011).**

| **№** | **Beggining** | **End** | **Cluster** | **%** |
| --- | --- | --- | --- | --- |
| 1 | 24377 | 27083 | 14-app | 100 |
| 2 | 164344 | 167094 | 15-app | 99,6 |
| 3 | 164344 | 166604 | 19-app | 87,4 |
| 4 | 204792 | 205744 | 16-app | 100 |
| 5 | 237923 | 240869 | c7 | 100 |
| 6 | 238739 | 240867 | c9 | 88,1 |
| 7 | 247858 | 249452 | c8 | 100 |
| 8 | 273064 | 274728 | 17-app | 99,9 |
| 9 | 273423 | 274506 | 18-app | 89,4 |

**2.** **The coordinates of 30 RTE-BovB/BTLTR1/RTE-BovB clusters localized in 12 structural genes (*kcne2, gart, tmem50b, il10rb, ifnar2, urb1, grik1, usp16, ltn1, cyyr1, app,* and *jam2*) in cattle (*Bos taurus*).**

The coordinates of 30 RTE-BovB/BTLTR1/RTE-BovB clusters localized in 12 structural genes (*kcne2, gart, tmem50b, il10rb, ifnar2, urb1, grik1, usp16, ltn1, cyyr1, app,* and *jam2*) in cattle (*Bos taurus*).

| № | Cluster | Beginning | End | Direction |
| --- | --- | --- | --- | --- |
| 1 | 1-*gart*-Bt | 1070392 | 1072259 | C/+/C |
| 2 | 2-*tmem50b*-Bt | 1148310 | 1151325 | C/+/C |
| 3 | 3-*il10rb*-Bt | 1367039 | 1368859 | C/+/C |
| 4 | 4-*il10rb*-Bt | 1376020 | 1377206 | C/+/C |
| 5 | 5-*ifnar2*-Bt | 1415386 | 1415979 | C/+/C |
| 6 | 6-*urb1*-Bt | 2231726 | 2233004 | C/+/C |
| 7 | 7-*grik1*-Bt | 5387766 | 5390683 | C/+/C |
| 8 | 8-*grik1*-Bt | 5453727 | 5454294 | C/+/C |
| 9 | 9-*grik1*-Bt | 5522536 | 5524480 | C/+/C |
| 10 | 10-*grik1*-Bt | 5595842 | 5597399 | C/+/C |
| 11 | 11-*grik1*-Bt | 5615401 | 5616958 | C/+/C |
| 12 | 12-*grik1*-Bt | 5710771 | 5712375 | C/+/C |
| 13 | 13-*ltn1*-Bt | 6460390 | 6462172 | C/+/C |
| 14 | 14-*app*-Bt | 9564838 | 9567544 | C/+/C |
| 15 | 15-*app*-Bt | 9704851 | 9707601 | C/+/C |
| 16 | 16-*app*-Bt | 9745299 | 9746121 | C/+/C |
| 17 | 17-*app*-Bt | 9813571 | 9815235 | C/+/C |
| 18 | 18-*app*-Bt | 9863980 | 9865070 | C/+/C |
| 19 | 19-*app*-Bt | 9885579 | 9887810 | C/+/C |
| 20 | 20-*jam2*-Bt | 10063930 | 10066275 | C/+/C |
| 21 | C1-*kcne2*-Bt | 465203 | 467062 | +/C/+ |
| 22 | C2-*grik1*-Bt | 5351839 | 5354773 | +/C/+ |
| 23 | C3-*grik1*-Bt | 5431052 | 5432565 | +/C/+ |
| 24 | C4-*grik1*-Bt | 5512950 | 5515185 | +/C/+ |
| 25 | C5-*usp16*-Bt | 6367127 | 6367525 | +/C/+ |
| 26 | C6-*cyyr1*-Bt | 9212127 | 9214071 | +/C/+ |
| 27 | C7-*app*-Bt | 9778430 | 9779999 | +/C/+ |
| 28 | C8-*app*-Bt | 9788365 | 9789959 | +/C/+ |
| 29 | C9-*app*-Bt | 9859229 | 9859982 | +/C/+ |
| 30 | C10-*jam2*-Bt | 10018863 | 10021029 | +/C/+ |
